# GMP-grade human neural progenitors delivered subretinally protect vision in rat model of retinal degeneration and survive in minipigs

**DOI:** 10.1186/s12967-023-04501-z

**Published:** 2023-09-25

**Authors:** Bin Lu, Pablo Avalos, Soshana Svendsen, Changqing Zhang, Laura Nocito, Melissa K. Jones, Cosmo Pieplow, Joshua Saylor, Sean Ghiam, Amanda Block, Michael Fernandez, Alexander V. Ljubimov, Kent Small, David Liao, Clive N. Svendsen, Shaomei Wang

**Affiliations:** 1 Board of Governors Regenerative Medicine Institute, Cedars-Sinai Medical Center, Los Angeles, CA 90048 USA; 2 David Geffen School of Medicine, University of California Los Angeles, Los Angeles, CA 90095 USA; 3 Macula& Retina Institute, Glendale, CA 91203 USA; 4 Retina Vitreous Associates Medical Group, Beverly Hills, CA 90211 USA

**Keywords:** Stem/neural progenitor cells, Retinal degeneration, Retinitis pigmentosa, Good manufacturing practice, Toxicology study, Animal models

## Abstract

**Background:**

Stem cell products are increasingly entering early stage clinical trials for treating retinal degeneration. The field is learning from experience about comparability of cells proposed for preclinical and clinical use. Without this, preclinical data supporting translation to a clinical study might not adequately reflect the performance of subsequent clinical-grade cells in patients.

**Methods:**

Research-grade human neural progenitor cells (hNPC) and clinical-grade hNPC (termed CNS10-NPC) were injected into the subretinal space of the Royal College of Surgeons (RCS) rat, a rodent model of retinal degeneration such as retinitis pigmentosa. An investigational new drug (IND)-enabling study with CNS10-NPC was performed in the same rodent model. Finally, surgical methodology for subretinal cell delivery in the clinic was optimized in a large animal model with Yucatan minipigs.

**Results:**

Both research-grade hNPC and clinical-grade hNPC can survive and provide functional and morphological protection in a dose-dependent fashion in RCS rats and the optimal cell dose was defined and used in IND-enabling studies. Grafted CNS10-NPC migrated from the injection site without differentiation into retinal cell phenotypes. Additionally, CNS10-NPC showed long-term survival, safety and efficacy in a good laboratory practice (GLP) toxicity and tumorigenicity study, with no observed cell overgrowth even at the maximum deliverable dose. Finally, using a large animal model with the Yucatan minipig, which has an eye size comparable to the human, we optimized the surgical methodology for subretinal cell delivery in the clinic.

**Conclusions:**

These extensive studies supported an approved IND and the translation of CNS10-NPC to an ongoing Phase 1/2a clinical trial (NCT04284293) for the treatment of retinitis pigmentosa.

**Graphical abstract:**

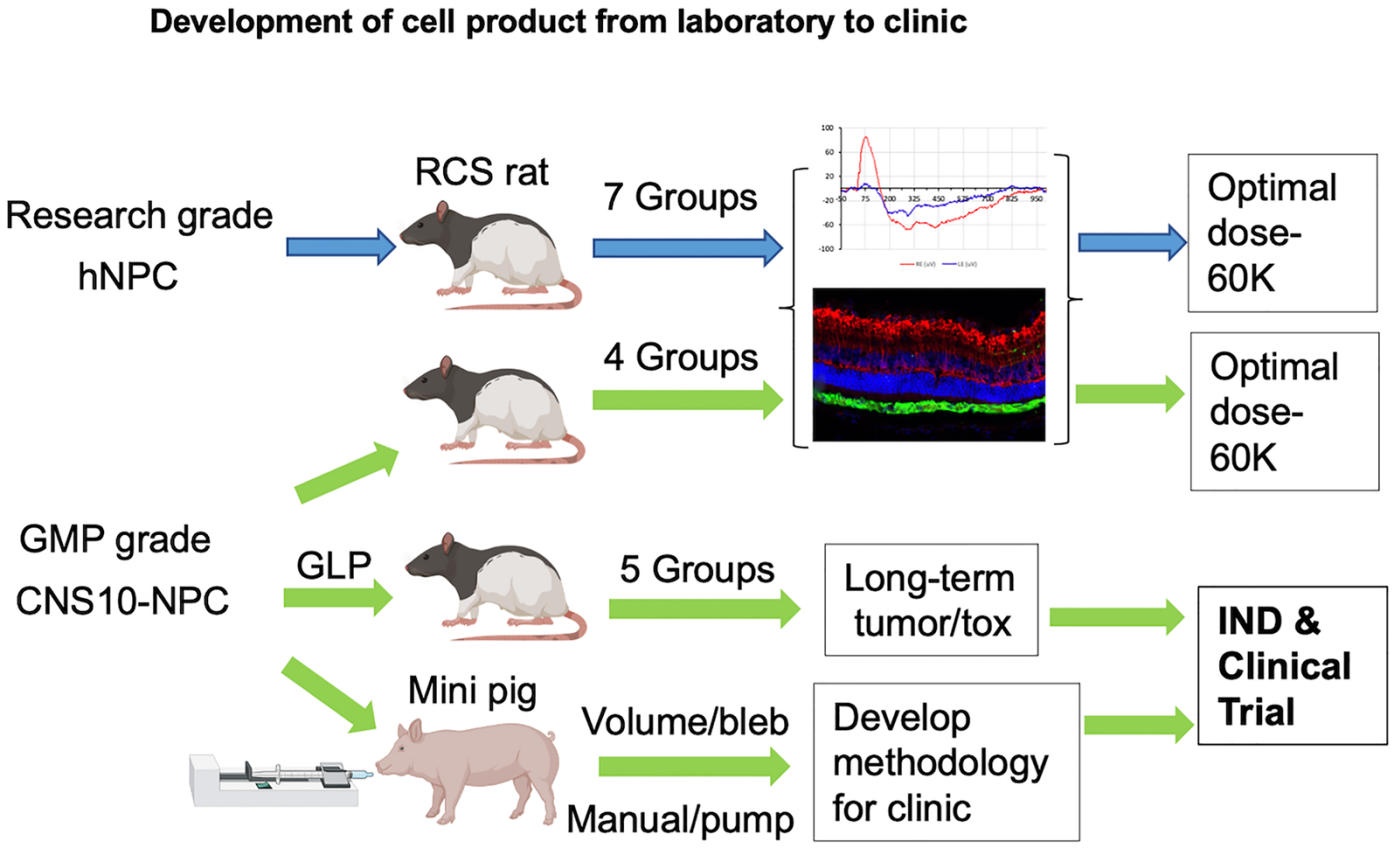

**Supplementary Information:**

The online version contains supplementary material available at 10.1186/s12967-023-04501-z.

## Background

Retinal degenerative diseases, such as retinitis pigmentosa (RP) and age-related macular degeneration (AMD) are the major causes of blindness in industrialized countries and affect millions of people worldwide [1–6]. These diseases, characterized by photoreceptor death as well as impairment of retinal pigment epithelial (RPE) cells, are caused by a variety of genetic defects and environmental factors [1, 7, 8]. Gene therapy for a single mutation, *RPE65*, has been approved by the United States Food and Drug Administration; however, long-term efficacy has not been consistently observed in clinical reports, especially at the late stage of intervention [9–11]. Unfortunately, treatment is still very limited for most patients with genetically complex or multifactorial retinal degeneration.

The application of stem/progenitor cells has shown promise for treating retinal degeneration, and many clinical trials for treating AMD and RP are ongoing in the United States and abroad. Two major strategies for these trials are cell replacement or preservation [12–15]. Transplanting healthy RPE cells to replace compromised and lost RPE cells has been studied extensively in preclinical and clinical settings for AMD [16–22]. Donor RPE cells have been derived from various sources [18, 21, 23–28]. However, long-term efficacy after RPE replacement remains to be established. A major problem is survival and integration of grafted RPE within the host Bruch’s membrane, which also undergoes progressive degeneration. In addition, degeneration creates a hostile microenvironment that affects grafted RPE cell survival and integration [29]. Photoreceptor replacement therapy is also an attractive idea, but remains challenging due to insufficient graft connectivity with host retinal neurons to re-establish retinal circuitry [14, 15, 30].

Another approach is to preserve existing photoreceptors and vision using supportive cells [31–36]. ReNeuron and jCyte have conducted Phase 2 clinical trials using retinal progenitor cells derived from fetal retina for the treatment of RP (clinicalTrials.gov: NCT02464436; NCT03073733). However, early this year, ReNeuron halted its cell-based therapy for RP after a Phase 2a trial that showed surgical complexity and limited efficacy. An alternative to a retinal progenitor cell type is human central nervous system derived neural stem/ progenitor cells. While a purified neural stem cell product (HuCNS-SC) from StemCells Inc provided some protection in an animal model of retinal degeneration [35], these cells are no longer commercially available. We have extensively assessed human neural progenitor cells (hNPC) derived from fetal forebrain or induced pluripotent stem cells. Using the well-established Royal College of Surgeons (RCS) rat model of retinal degeneration, we have shown that subretinal delivery of 20,000 (20 K) to 40 K hNPC can survive long-term, migrate from the injection site and offer dramatic preservation of photoreceptors and vision [31, 37–39] through various mechanisms of action, including to regulate the immune response by inhibiting microglia activation; promote antioxidant effects; release pro-survival factors by upregulating Nrf2 [32, 40]; prevent outer segment accumulation by phagocytosis [38] and release protective trophic factors including FGF-2, IGF-1 [31]. hNPC have low expression of MHCs [41, 42] and hence low immunogenicity, which can enable long-term survival after engraftment even in the absence of immunosuppression. Collectively, these preclinical studies support moving hNPC transplants for RP into the clinic.

As stem cell products are increasingly entering early stage clinical trials, the field is learning from experience about how stem cell products may be best assessed for safety and efficacy. In two separate studies, a research-grade human neural stem cell product, HuCNS-SC, showed good efficacy for central nervous system repair in two different animal models, yet the closely related clinical-grade product did not have evident efficacy [43–45], presumably due to manufacturing differences. This emphasizes the need for increased cell characterization to determine comparability of cells proposed for preclinical and clinical use. Without this, preclinical data supporting translation to a clinical study might not adequately reflect the performance of subsequent clinical-grade cells in patients.

Here we first present a dose ranging study that showed 60 K hNPC was the optimal dose to slow degeneration in RCS rats. We then assessed the functionality of clinical-grade cells (named CNS10-NPC) that also preserved photoreceptors and vision in the RCS rat at the same optimal dose of the 60 K. CNS10-NPC survived and migrated from the injection site, and remained as neural progenitor cells or differentiated into astrocytes. A long-term tumorigenicity and toxicity good laboratory practice (GLP) study showed cell survival, with no cell overgrowth, even with the maximum feasible dose up to 180 days post-transplantation. Finally, the methodology to deliver CNS10-NPC was optimized in a large animal model, which showed CNS10-NPC survival in a layer covering an extensive area of the subretinal space of Yucatan minipigs. These critical Investigational new drug (IND)-enabling studies defined the optimal cell dose as well as confirmed the function, safety and delivery of clinical-grade CNS10-NPC, which paved the way for an ongoing Phase 1/2a clinical trial (NCT04284293) using this cell product to treat RP.

## Materials and methods

### Study design

#### Cell production

Good manufacturing practice (cGMP) generation and expansion of human neural progenitors has been previously described [46]. A human fetal cortical sample, termed G010, was collected from a single donor and expanded in epidermal growth factor and fibroblast growth factor-2 for 14 passages as free-floating aggregates. The aggregates were passaged using a chopping method [47], and transferred into a cGMP facility (WCBF, Waisman Center, Madison, WI) for 5 further passages before cryopreservation as the Master Cell Bank (MCB), which underwent full adventitious agent testing. Vials from the MCB were then used to create both a non-GMP research-grade cell lot and a GMP clinical-grade cell lot (termed CNS10-NPC). Briefly, MCB vials were thawed and expanded under process-comparable conditions for an additional 5 passages for both cell lots that were then cryopresered. The process-comparable research-grade cell line (hNPC) and clinical-grade CNS10-NPC were used in these studies under the Stem Cell Research Oversight Committee (Pro00025772).

#### Cell preparation

Cells did not undergo propagation in culture for these studies, but rather hNPC or CNS10-NPC vials were thawed and cells were suspended in 10 mL wash media and collected by centrifugation. Cell pellets were resuspended in transplantation medium (termed vehicle). A viability count was performed using a hemocytometer and Trypan blue exclusion. Cells were then pelleted again and resuspended in vehicle at the required concentration. Cells were stored overnight at 4 °C, and cell viability counts were repeated before injection to confirm concentration and viability above 70%. Cells were resuspended by flipping the tube prior to loading into the glass micropipette immediately before each injection.

#### Rat experiments

Experiments used pigmented dystrophic male and female RCS rats (*rdy*^+^*, p*^+^). In the RCS rat model of retinal degeneration, RPE cells cannot phagocytose shed photoreceptor outer segment (POS) due to a mutation in the transmembrane proto-oncogene tyrosine-protein kinase MER (*MertK*) [48, 49]. The build-up of POS blocks nutrients from the choroid to photoreceptors, leading to photoreceptor death. Rats received a unilateral subretinal injection of hNPC or CNS10-NPC in 2 μl of vehicle at postnatal day (P) 21–23 based on our published protocol [37, 39] and standard operating procedures. Optimal dose was determined using research-grade hNPC at five doses (6,000 (6 K), 20 K, 60 K, 200 K, 400 K) (Additional file 1: Table S1A). Based on our published studies showing that injection of 20 K showed efficacy [31, 37], the dose range selection used 6 K as a low dose and used 60 K and 200 K as higher doses. The 400 K dose was used to test the maximum deliverable dose using our delivery device. Subsequently efficacy of clinical-grade cells was confirmed with CNS10-NPC at three doses (6 K, 60 K, 400 K) (Additional file 1: Table S1B). In both experiments, control rats received vehicle, and fellow eyes served as untreated controls. Animals were housed and maintained at the Cedars-Sinai Medical Center, Department of Comparative Medicine vivarium and animal studies were approved and supervised by the Cedars-Sinai Institutional Animal Care and Use Committee (IACUC 3801). Animals were euthanized around P90. The long-term GLP tumorigenicity and toxicology study was performed by the Contract Research Organization Absorption Systems (San Diego, CA) under their study number 16C302Q1G109. RCS rats received CNS10-NPC at 6 K, 60 K, or 400 K, or vehicle (Additional file 1: Table S1C1). Animals were euthanized at approximately days 7, 30 and 180 post-surgery, which correlates to approximately P30, 52 and P202 (Additional file 1: Table S1C2). All animals received daily intraperitoneal injection of dexamethasone for 2 weeks (1.6 mg/kg per day) after surgery and were immunosuppressed by ad libitum oral cyclosporine A administered in drinking water (210 mg/l) throughout the study. All animals were treated in accordance with the ARVO Statement for the Use of Animals in Ophthalmic and Vision Research.

#### Large animal experiments

The Yucatan minipig eye size is comparable to the human [50], and 13 minipigs were used to optimize cell delivery for the clinic (Table 1). Briefly, a lateral canthotomy incision (about one inch) was made, and an eyelid speculum and sutures were placed to retract the eyelids. The following maneuvers were performed under direct visualization via an operating microscope. The central and posterior vitreous were removed together with the posterior hyaloid membrane by means of a localized 3-port 23-gauge pars plana vitrectomy, using a standard vitrectomy unit (i.e., Constellation, Alcon, Fort Worth, TX). A 250µL Hamilton syringe (1725TLLX250SYR) was connected to extension tubing (Medex 536020) and a subretinal PolyTip® cannula (MedOne 3219). The syringe, tubing and cannula were primed with vehicle prior to cell loading. In some cases, the syringe was attached to a positive displacement microinjector pump (MINJ-PD, Tritech Research Inc., los Angeles, CA). The polytip of the cannula was inserted into the subretinal space and cell suspension was delivered, with or without prior bleb formation. A pars plana partial vitrectomy with non-valved 23 g trocars was performed followed by injection of CNS10-NPC into the subretinal space either by manual or automatic injection. Manual injection comprised of a 25 g/38 g polytip fixed-length cannula (MedOne); extension tubing (Medstream); and a 1 mL syringe. The automatic injection system comprised of a 25 g/38 g polytip cannula, extension tubing and a positive displacement microinjection pump with a 250 µL Hamilton Syringe (Tritech Research). After injection, pigs were either euthanized under anesthesia or recovered from anesthesia and kept alive for up to 7 days and then euthanized. In survival studies, pigs were immunosuppressed intraoperatively with tacrolimus at 0.1 mg/kg and a 125 mg bolus of methylprednisolone, followed by oral tacrolimus twice a day mixed in with food, which was started two days prior to cell injection until euthanasia. The study design and animal usage were reviewed and approved by the Institutional Animal Care and Use Committee (IACUC 6570) for compliance with regulations prior to study initiation. Animal welfare for this study was in compliance with the Guide for the Care and Use of Laboratory Animals and the Cedars-Sinai Department of Comparative Medicine Veterinary staff.

**Table 1:** Methodology development for subretinal delivery of CNS10-NPC in minipigs

Experiment objective	Variable tested	Survival (Days)	Cell concentration and volume	# Pigs	# Eyes
Manual vs. automatic delivery	Manual	Non-survival	10,000 cells/ µl in 50µL	2	3
	Manual	7	10,000 cells/ µl in 50µL	4	5
	Automatic	7	50,000 cells/ µl in 50µL	1	2
Cell concentration and volume	Automatic	6	10,000 cells/ µl in 10µL	2	4
Bleb requirement	No Bleb	Non-survival	10,000 cells/ µl in 50µL	1	2
	Air Bleb	Non-survival	10,000 cells/ µl in 50µL	1	2
	Air Bleb	7	10,000 cells/ µl in 50µL	2	4

#### Visual function tests

All rats were tested by optokinetic response (OKR) and electroretinography (ERG) at P60 and P90, according to our published protocols [31, 51, 52]. For the long-term study, all animals were tested by OKR at 180 days post-surgery. OKR offers noninvasive screening to detect visual acuity. Briefly, the test began after the rat was placed on the pedestal,  and tracking is defined by smooth, reflexive head movement in the same direction of the rotation. Once detected, the spatial frequency would be increased until no more successful tracking could be observed. Two observers blind to the treatments were present to record and verify the tracking of the rodent. ERG measures the average retinal responses to light stimulation, to provide a gross measure of retinal activity and indicate the relative function of rods and cones. Before ERG, animals were dark adapted overnight (> 12 h) and then prepared in a dim, red light. Under anesthesia, the head and body were secured to the testing device. Topical anesthesia was applied to prevent reflexive movements in the cornea and saline was used for hydration and the ability to attach the recording electrode. Care was taken to maintain the electrode placement in the same position in all animals. The eye was stimulated with full-field light flashes. Corneal potentials were recorded with the amplifier connected to the electrode. Flash presentations were controlled with a computer program. The responses were averaged for 20 stimulus presentations.

#### Spectral domain optical coherence tomography (SD-OCT)

This is a noninvasive, trans-pupillary method that provides in vivo cross-sectional images of the retinal lamination. RCS rats and minipigs were anesthetized, pupils were dilated using Tropicamide ophthalmic and Phenylephrine hydrochloride ophthalmic solutions (Akorn, Lake Forest, IL). Ofloxacin ophthalmic (Akorn) drops were applied to the eyes to prevent infection. The images of retinal cross sections were selected from a 3D high-resolution image captured by the Envisu R2210 image guided SD-OCT system (Bioptigen, Morrisville, NC).

#### Histology

At the end of the experiment, eyes from RCS rats and Yucatan minipigs were removed and immersed in 4% paraformaldehyde for one hour (rat) or overnight (minipig), then infiltrated with 10% and 20% sucrose for one hour each, and then overnight at 4 °C in 30% sucrose. The corneas and lenses were removed, and the eyes were embedded in optimal cutting temperature compound (OCT. Sakura Finetek USA, Torrance, CA). Cryostat retinal Sects. (10 µm for rat, 15 µm for minipig) were collected in 5 series according to our previous protocol [38]. One slide from every five slides was stained with cresyl violet (CV) and the remaining slides were stored at − 80 °C for antibody staining. CV-stained images were taken with a regular bright field microscope.

#### Immunostaining

Immunofluorescent staining of retinal sections was performed with primary antibodies listed in (Additional file 2: Table S2) using our published protocols [38, 51]. Anti-mouse or rabbit secondary antibodies conjugated to Alexa Fluor-488 or Alexa Fluor-568 (Thermo Fisher Scientific, Waltham, MA) were used and counterstained with 49,69-diamidino-2-phenylindole (DAPI). Images were taken with a confocal microscope (Eclipse C1si; Nikon Instruments, Inc., Melville, NY). For non-fluorescent immunohistochemistry, primary antibodies were incubated overnight, then biotinylated mouse and rabbit secondary antibodies were incubated for one hour at room temperature. After incubation with Avidin/biotin complex (ABC) solution for one hour, retinal sections were incubated with nickel-intensified diaminobenzidine (DAB). Retinal sections were counterstained with CV, dehydrated in alcohols and cleared in xylene before mounting. Images were taken with a light microscope. In the GLP tumor/ toxicology study, tissue was collected by Absorption Systems (San Diego, CA) and processed by Inotiv (formerly, Seventh Wave, Missouri). For Yucatan minipig retinal sections, human-specific nuclear marker and nestin were used to identify donor cells.

#### Quantification of outer nuclear layer (ONL) protection

Five CV-stained sections with preserved ONL (5 sections/eye, 3 eyes/group) were selected to quantify ONL preservation. Retinal montage images were prepared for measuring the length of preserved ONL against the whole retinal length by Java-based image processing software (ImageJ; National Institutes of Health, Bethesda, MD). The measurement started at the area with more than two layers of ONL around the injection side. There was only a single layer of ONL remaining in untreated retina at the time when tissue was harvested.

#### Quantification of CNS10-NPC survival

Five retinal sections from representative slides with grafts stained with human-specific nuclear marker MAB1281 (5 sections/eye, 6 eyes/group) were selected to quantify donor cell survival. MAB1281-positive donor cells were counted manually. The cell number in non-assessed sections was determined by linear interpolation between known cell numbers in the assessed sections, thereby calculating an estimated total cell number per retina.

#### Statistical analysis

Data were analyzed with GraphPad Prism (GraphPad Software, Inc., La Jolla, CA). Length of ONL preservation, visual acuity by OKR and b-wave amplitudes were analyzed by one-way ANOVA with Tukey’s test. *P* values ≤ 0.05 were considered significant. Error bars indicate standard error of the mean (SEM).

## Results

### Determination of optimal cell dose

We have previously established that hNPC can preserve vision and photoreceptors in rodent models of retinal degeneration [31, 32, 37, 39, 40]. However, as the optimal cell dose was not systematically assessed, we first established this using research-grade hNPC. Cells were delivered to the subretinal space in RCS rats at five escalating doses of either vehicle; 6 K, 20 K, 60 K, 200 K and 400 K cells; and the untreated contralateral eye served as a non-treated control. Based on OKR analysis, visual acuity was preserved at both P60 and P90 timepoints (Additional file 4: Fig. S1A, B), with the 60 K dose offering significantly higher visual acuity compared to the lower doses, vehicle- and untreated controls. Doses of 60 K, 200 K and 400 K were not significantly different at either timepoint, showing the maximum effect can be achieved at 60 K. While b-wave amplitude declined with time, ERG analysis demonstrated that eyes treated with hNPC had significantly preserved b-wave amplitudes at both P60 and P90 timepoints (Additional file 4: Fig. S1C, D). The 6 K dose was similar to vehicle treatment. A dose of 20 K significantly increased b-wave over the controls at P60, though preservation was most evident in groups with doses of 60 K and 200 K. The maximum feasible dose of 400 K did not show a further increase in viable b-waves.

SD-OCT analysis showed that the ONL is visible at P60 and there were observed clusters of hNPC in all treated groups except the lowest dose (Additional file 4: Fig. S1E). By P90, the ONL is no longer visible in rats treated with vehicle or the lowest dose. In contrast, the ONL was protected in regions near engrafted cells, which appeared flattened out over time. Further evaluation of CV-stained sections at P90 showed that RCS rats receiving vehicle or the 6 K dose had only 2–3 layers or 3–4 layers of ONL remaining at the injection site, respectively (Additional file 5: Fig. S2A). In contrast, the dose of 20 K offered consistent ONL preservation and higher doses (60 K–400 K) had a much broader area of ONL preservation (6–8 layers of photoreceptors). Quantification of the length of preserved ONL over the total length of retinal section showed that the 60 K group had a significantly higher percentage of ONL preservation than the 20 K group, while the differences among 60–400 K groups were not significant (Additional file 5: Fig. S2B). Further, cone arrestin antibody staining revealed cell treatment preserved cone morphology with axons, pedicles and density (Additional file 5: Fig. S2C) compared with degenerative cones in untreated retinas. Collectively, this dose ranging study determined that 60 K is the optimal dose to provide protection against functional loss, and that higher dose does not produce better protection. While the optimal dose of 60 K for hNPC is lower than the dose used in some other studies [17, 53, 54], this is not unexpected given variable functionality between cell types.

### Clinical-grade CNS10-NPC protect vision and photoreceptors

**Fig. 1:**
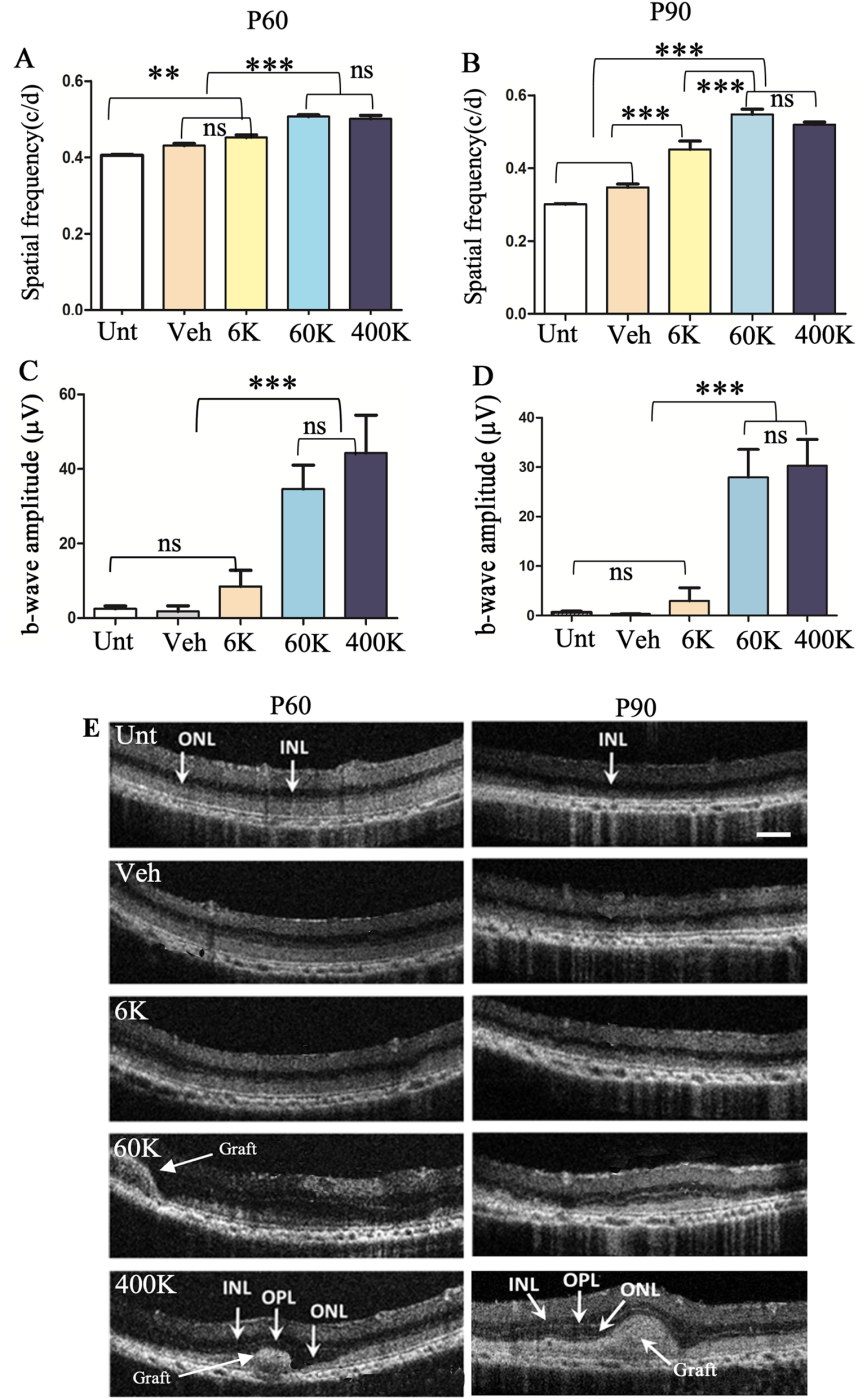
CNS10-NPC preserve vision in a dose–response fashion. (**A** and **B**) OKR shows CNS10-NPC protect visual acuity in a dose-dependent manner, with 60 K being the optimal dose (n = 10/treatment). Visual acuity remained unchanged over time from 60K and 400 K groups. (**C** and **D**) ERG reveals the optimal dose offers significantly higher b-wave amplitude compared with the low dose and control groups. There is no significant difference between 60K and 400 K groups. (**E**) SD-OCT was performed at P60 and P90 following cell or vehicle injection at P21-23. At P60, ONL is visible from all the groups, but is clearly thicker in the 60 K and 400K  CNS10-NPC groups. Cell clusters (Arrows in 60 K and 400 K) were detected in retinas with 60 K and 400 K at P60 associated with thicker ONL, and these clusters largely flattened out at P90 in the 60 K but not 400 K group. The ONL in lower dose and control groups was no longer visible, while in 60 K or higher dose-treated groups, ONL was clearly visible at P90. Data are represented as mean ± SEM. One-way ANOVA with Tukey’s test was used for multiple comparisons. ** p < 0.01; *** p < 0.0001; ns: no significance. Scale bar = 200 µm. *INL* inner nuclear layer, *ONL* outer nuclear layer, *OP﻿L* outer plexiform layer

While preclinical data using research-grade cells is promising, these cells may not adequately reflect the performance of subsequent batches of clinical-grade cells intended for use in patients [43–45]. For translation to the clinic, we next assessed GMP clinical-grade neural progenitor cells, termed CNS10-NPC, at three escalating doses of 6 K, 60 K and 400 K cells as well as vehicle and untreated controls. Corroborating the dose response study with hNPC, CNS10-NPC preserved visual acuity in a dose-dependent fashion at both P60 and P90 (Fig. 1A, B) timepoints. The dose of 60 K offered significantly higher visual acuity over the lower dose and vehicle and untreated controls. There was no significant difference between 60 and 400 K, indicating 60 K was the optimal dose. While b-wave amplitude again declined over time, ERG analysis showed that CNS10-NPC led to preserved b-wave amplitudes at both P60 and P90 timepoints, in a dose-dependent manner (Fig. 1C, D). The optimal dose of 60 K significantly preserved b-wave over the controls and the low dose at both timepoints, and the high dose of 400 K did not offer extra benefit.

**Fig. 2:**
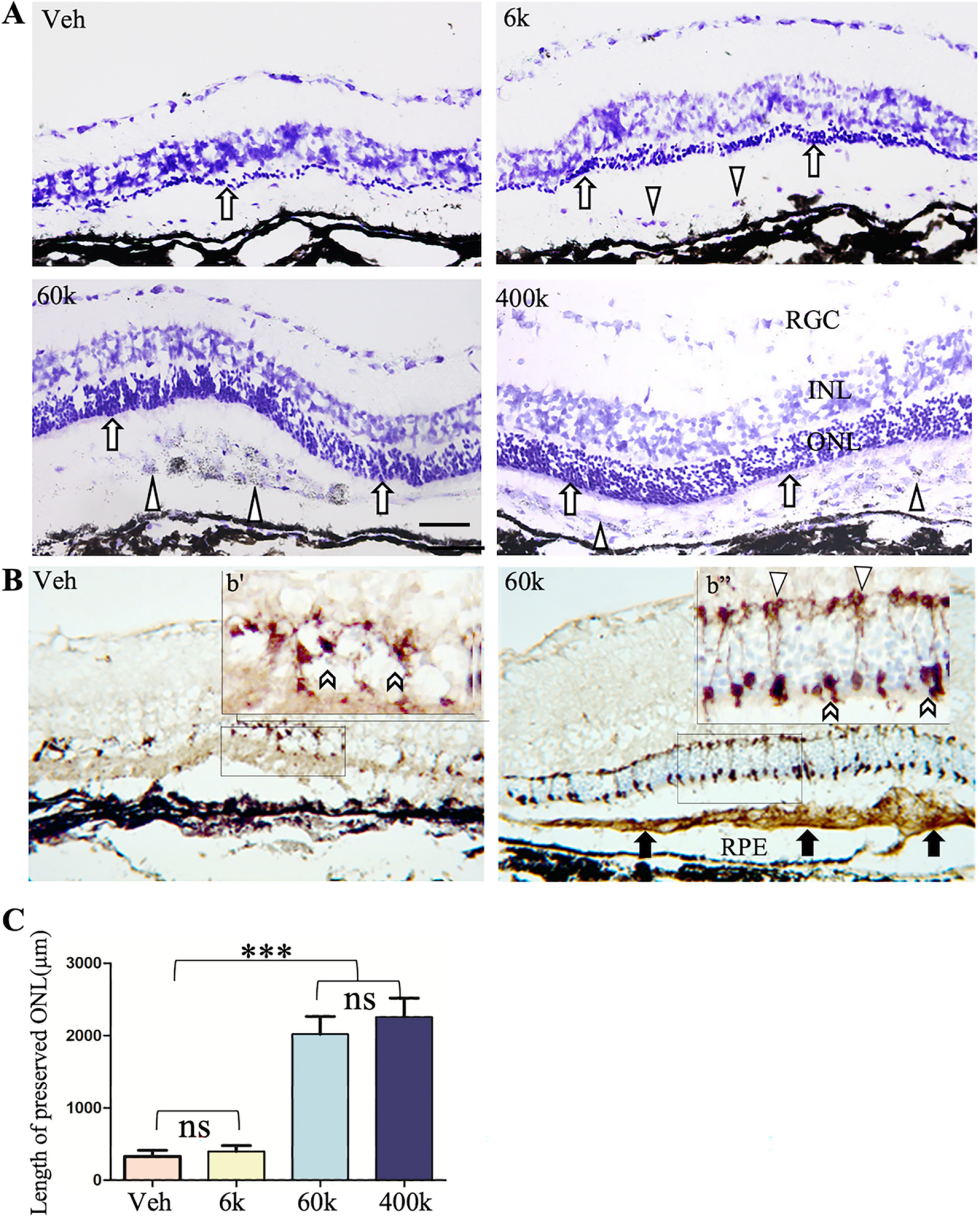
CNS10-NPC protect photoreceptors in a dose–response fashion. (**A**) Retinal P90 sections stained with cresyl violet show extensive photoreceptor preservation with 60 K and 400 K doses compared with other groups. Photoreceptor protection is associated with donor cell distribution (Triangles in 60 K and 400 K), with 6–8 layers of photoreceptors (Arrows) preserved, compared with 2–3 layers (Arrows) in 6 K group, some donor cells contained pigmental granules (Triangles in 60 K and 400 K). (**B**) Retinal sections from vehicle and 60 K treated groups were stained with Stem 121 (brown color, arrows in 60 K) and cone arrestin (dark purple, chevron showing cones in b’ and b’’, downward triangles showing cone pedicles in b’’). Cone photoreceptors from 60 K group with segments (Chevrons) and pedicles (Triangles) were observed compared with vehicle treatment. b’ and b’’ are high power images from the corresponding outlines. (**C**) The length of preserved ONL (more than 2 nuclei thickness) against the whole retinal length by ImageJ showed 60 K and 400 K groups had significantly greater ONL protection than other groups. Data are represented as mean ± SEM. One-way ANOVA with Tukey’s test was used for multiple comparisons. *** p < 0.001; ns: no significance. Scale bar = 50 μm. *INL* inner nuclear layer, *ONL* outer nuclear layer, *RGC* ganglion cell layer, *RPE* retinal pigment epithelium

We next determined whether the ability of CNS10-NPC to protect visual function correlated with retinal protection. As with hNPC, SD-OCT analysis showed ONL preservation in retinas treated with CNS10-NPC at the higher dose (60 K and 400 K) (Fig. 1E). Cell clusters were observed in high dose groups at P60, which largely flattened out in the 60 K dose group by P90 (Fig. 1E). ONL was reduced in thickness in lower dose and control retinas from P60 to P90. Furthermore, retinal histological examination revealed consistent ONL preservation (6–8 layers) in high dose-treated retinas (60 K and 400 K) compared with low dose-treated and control retinas (Fig. 2A). As preserving cone function is critical for patients with RP, we next assessed retinal lamination and cone photoreceptors following treatment with CNS10-NPC. Retinal sections stained with cresyl violet show extensive photoreceptor preservation with 60 K and 400 K groups, compared with vehicle- and 6 K-treated retinas (Fig. 2A). Further, cone photoreceptors were stained with a cone arrestin antibody followed by intensified nickel (purple), and grafted CNS10-NPC were identified by the human-specific cytoplasmic marker, Stem 121, labeled by DAB. CNS10-NPC survived and, critically, the treated retina had cones with pedicles, axons and inner and outer segments (Fig. 2B, 60 K vs. 6 K) compared to the vehicle-treated group with a disorganized, fragmented cone profile. Quantifying the length of preserved ONL (> 2 cell thick) by Image J showed that the high dose groups (60 K and 400 K) offered significantly better ONL preservation than other groups (Fig. 2C). However, the 400 K dose did not significantly further increase ONL preservation compared to 60 K. Collectively, these results demonstrate that CNS10-NPC, at the optimal dose of 60 K, provide functional and morphological protection after a single subretinal injection in the RCS rat.

### In vivo characterization of CNS10-NPC

**Fig. 3:**
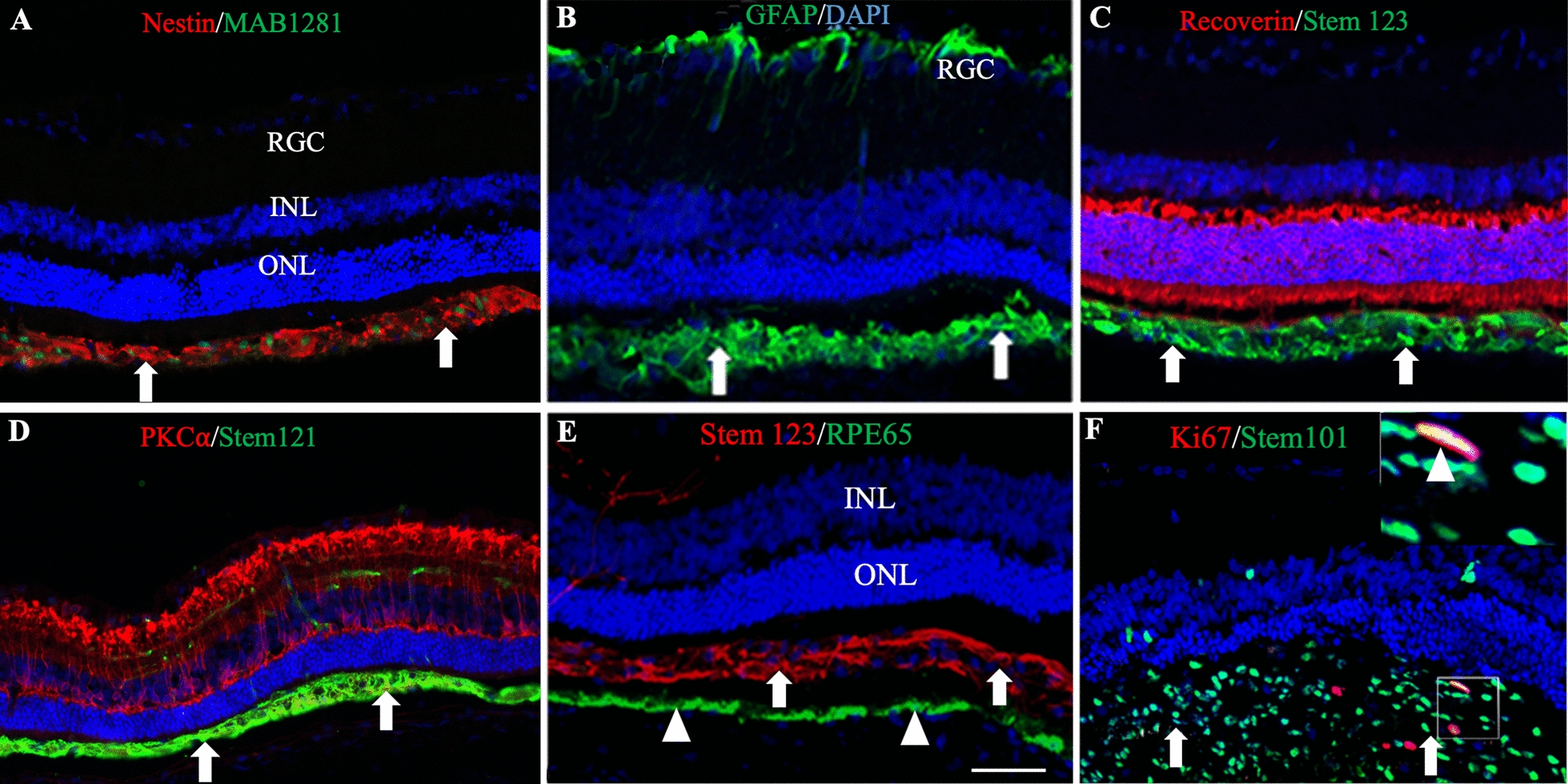
CNS10-NPC in vivo characterization. **A** and **B**: Retinal P90 sections from 60 K group double stained with human nuclear marker, MAB1281, and nestin (**A**) and GFAP (**B**) show donor cells (Arrows) remained as neural progenitor cells and differentiated into astrocytes. It is noted that photoreceptor preservation is associated with donor cell distribution. Host astrocytes in RGC layer (**B**) also stained positive for GFAP. **C–E**: Retinal sections from 60 K (**C**) and 400 K (**D** and **E**) groups that were co-stained with human-specific markers Stem 123 and Stem 121 (Arrows) along with the photoreceptor marker recoverin (Red in **C**), rod bipolar cell marker PKCα (Red in **D** and retinal pigment epithelial marker RPE65 Green in **E**). No double positive staining was detected, indicating donor cells did not differentiate into retinal cells. (**F**) Retinal sections from the 400 K group were double stained with human nuclear marker Stem 101 (Green, arrows) and cell proliferation marker Ki67 (Red), which showed only minimal cells with double positive staining. Insert is high power image of the outline showing double-stained nuclei (Triangle). Scale bar = 50 μm. *INL* inner nuclear layer, *IPL* inner plexiform layer, *ONL* outer nuclear layer, *OPL* outer plexiform layer, *RGC* ganglion cell layer, *RPE* retinal pigment epithelium

In order to characterize transplanted CNS10-NPC, immunohistochemistry was performed on retinal sections at P90 from rats receiving 60 K and 400 K. A human-specific nuclear marker (MAB1281) showed that CNS10-NPC formed a layer next to host photoreceptors, with a subset of cells staining as nestin-positive neural progenitors (Fig. 3A). CNS10-NPC survival at P90 was quantified on retinal sections stained with MAB1281 (Additional file 3: Table S3A). The total number of positive donor cells in representative sections was counted manually in the subretinal space and inner retina. Of note, the 60 K optimal dose group shows a higher donor cell survival rate than the 400 K group. Staining with an antibody against glial fibrillary acidic protein (GFAP) showed some CNS10-NPC had differentiated into astrocytes (Fig. 3B). As published for hNPC [31, 37], CNS10-NPC migrated into the inner retina but did not differentiate into retinal cells, based on no observed co-labeling for human astrocyte-specific Stem 123-labeled donor cells with recoverin-positive photoreceptors, PKCα-positive rod bipolar cells or RPE65-positive RPE cells (Fig. 3C–E). Collectively, these results indicate that grafted CNS10-NPC remain as neural progenitor cells or differentiate into astrocytes but not into retinal cell types. These findings, along with our published long-term studies [31, 37], demonstrate that the neural progenitor cells offer retinal protection not via a regenerative mechanism through becoming a retinal cell type, but via various pathways offering protection such as regulating host immune response, promoting antioxidant effects and releasing trophic factors. Finally, a main safety concern with stem/progenitor cells is continued proliferation and hence tumor formation in vivo. Following delivery of the maximum feasible dose, retinal sections double stained with the human-specific nuclear marker, Stem 101, and cell division marker Ki67 showed only limited double-positive cells, which occurred as sparse individual cells instead of a proliferative mass (Fig. 3F). This is consistent with our previous results from hNPC grafts in RCS rats [37].

### CNS10-NPC show long-term safety and efficacy

**Fig. 4:**
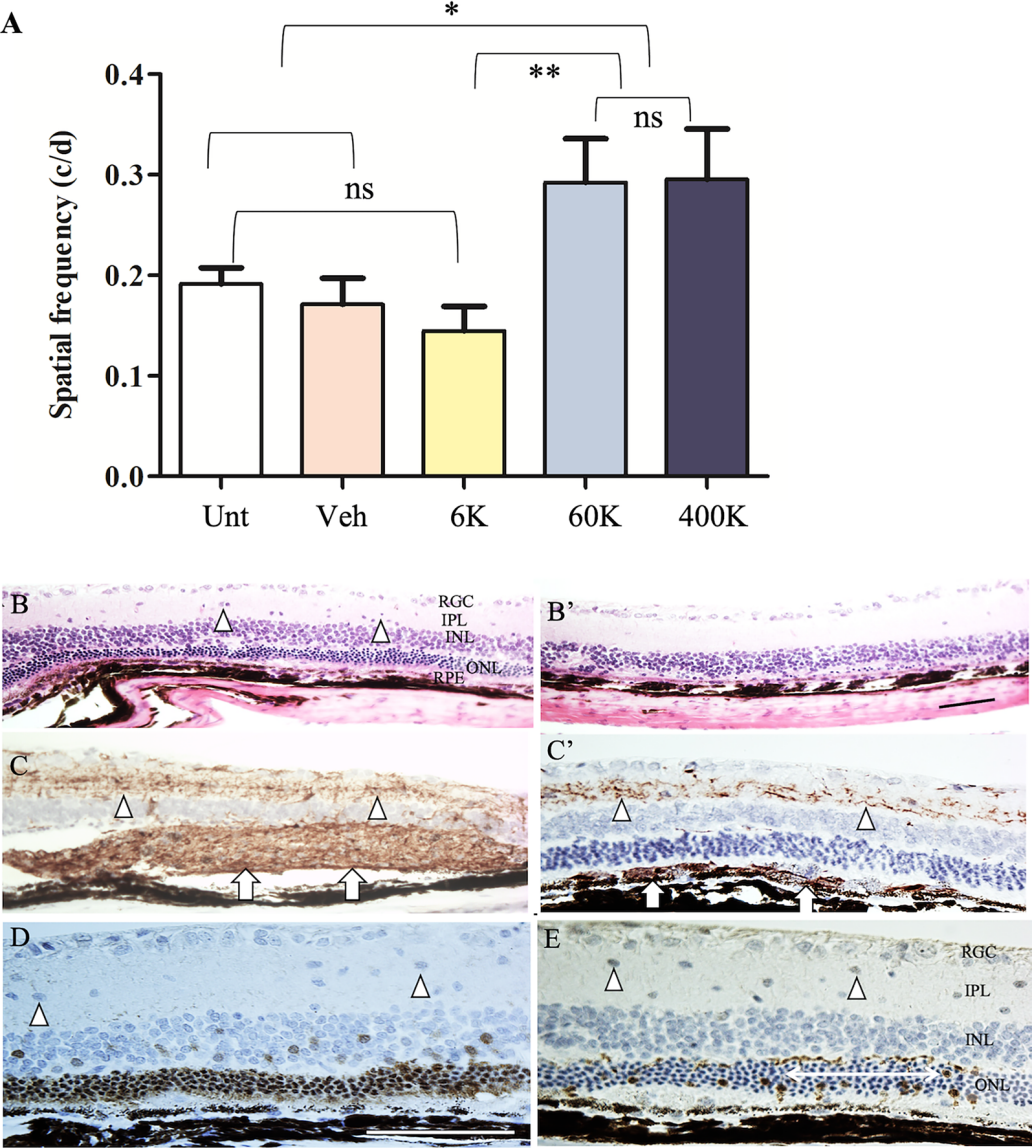
Long-term vision and photoreceptor protection and donor cell survival from GLP study. (**A**) OKR measurement shows that visual acuity in high dose groups was significantly better than other groups. It is noted that there are variations among high cell dose groups, which is due to cornea opacity or lens cloudy post-surgery. (**B**) Hematoxylin and eosin stained retinal section from 400 K group shows 3–5 layers of photoreceptors at the graft core and only sparsely distributed photoreceptors distal from the graft **B’** at 6 months post-injection. Triangles in **B** indicate donor cells migrating to the inner retina. (**C** and **C**’ Retinal sections stained with human-specific antibody Stem 121 show that grafted CNS10-NPC formed a lump (Arrows in **C**) and layers (Arrows in **C’**) of cells in the subretinal space, and some cells migrated into the inner retina (triangles in **C** and **C’**). (**D** and **E**) Retinal sections stained with photoreceptor marker recoverin and cone marker, cone arrestin, show 3–5 layers of photoreceptors and cone preservation (Double arrow line in **E**), triangles indicating donor cells migrating to the inner retina. Data are represented as mean ± SEM. One-way ANOVA with Tukey’s test was used for multiple comparisons. *p < 0.05; ** p < 0.01; ns: no significance. Scale bar = 75 μm. *INL* inner nuclear layer, *IPL* inner plexiform layer, *ONL* outer nuclear layer, *OPL* outer plexiform layer, *RGC* ganglion cell layer, *RPE* retinal pigment epithelium

Having confirmed the functional efficacy of CNS10-NPC, it was next critical to confirm long-term safety. In a GLP tumorigenicity and toxicology study (Absorption Systems, CA), RCS rats received subretinal injections of CNS10-NPC at three doses (6 K, 60 K, and 400 K cells), and visual acuity measured by OKR was performed from all the experimental groups. OKR showed both 60 K and 400 K groups had significantly higher visual acuity compared with other groups 180 days post-injection (Fig. 4A), with no difference between the two higher cell doses. Retinal histology was examined at days 7, 30 and 180 post-injection. No CNS10-NPC-related toxicity or tumorigenicity findings were present in the eyes or optic nerves or brain and other tissues and organs at any timepoint. Injection of CNS10-NPC did not affect animals’ physiology, and no adverse responses were detected in body weight and weight from multiple organs including the brain, heart, lung, liver and kidney (Additional file 6: Fig. S3) and blood chemistry (data not shown). Retinal sections from the 400 K group at 180 days post-injection stained with H&E showed 3–5 layers of preserved ONL in the cell-treated retina, compared with a sparsely distributed photoreceptors in an area distant from the injection site (Fig. 4B vs. B’). Immunohistochemistry with human-specific Stem 121 revealed that donor cells survived up to 6 months and formed as a lump of cells or as layers of cells in the subretinal space (Fig. 4C and C’). Donor cells migrating into inner retina were also observed. CNS10-NPC were detected at the three doses at the different survival timepoints (Additional file 3: Table S3B), and it is noted that donor cells were detected in 11 of 15 eyes examined in 400 K group. Further, antibody staining of recoverin and cone arrestin showed preserved photoreceptors and cones (Fig. 4D and E). In addition, Ki67 staining on retinal sections from the optimal 60 K dose group showed limited positive cells at 7 and 30 days post-injection, and no positive cells at 180 days (Additional file 3: Table S3C), demonstrating that clinical-grade CNS10-NPC product were not proliferative and were non-tumorigenic.

### CNS10-NPC survive and distribute extensively in the minipig

The Yucatan minipig eye size is comparable to the human [50] and hence was used to optimize the surgical methodology for subretinal cell delivery in the clinic (Table 1). Manual injection was first tested by delivering CNS10-NPC either unilaterally or bilaterally into the subretinal space, at 10 K cells/µL in 50 µL, to pigs at a non-survival timepoint (n = 2) or a 7day post-injection survival timepoint (n = 4). Histological evaluation of collected eyes (n = 8 total) revealed CNS10-NPC in 1 of the 3 eyes in the non-survival pigs and 1 of the 5 eyes in the 7 day survival pigs. Manual injection does not permit specific control of the injection rate, which could result in cell reflux, and introduces variability in volume delivered between surgeries. As such, a microinjection pump for cell delivery was used to assess cell concentration and volume. A minipig (n = 1) received a bilateral injection of 10 K cells/µL in 50 µL with a 7 day survival and minipigs (n = 2) received 50 K cells/µL in 10 µL with a 6 day survival delivered bilaterally (500 K cells total in all cases). Histological examination revealed CNS10-NPC in 1 of 2 eyes from the 10 K cells/µL in 50 µL design and no donor cells were detected in the 50 K cells/µL in 10 µL design. Therefore, 10 K cells/µL in 50 µL was used in subsequent optimization experiments with an automatic pump injection system.

Subretinal injection creates a retinal detachment or “bleb,” which can be formed by direct injection of cell suspension or by injecting fluid or air prior to cell infusion. In the above experiments, transplantation media (75–120 µL) was injected via a cannula into the subretinal space over 10–30 s for bleb formation. The media delivery cannula was then removed and a cell delivery cannula was introduced through the same retinotomy. The lack of cells in most eyes could be due to an enlarged retinotomy caused by the insertion of the second cannula and, potentially, increased pressure inside of the bleb and hence reflux of the cells. To eliminate the 2-cannula approach, cells were next delivered bilaterally into the subretinal space via automatic injection with one cannula and no bleb, at a non-survival timepoint (n = 1). Microscopic evaluation during delivery revealed cell reflux occurred with both eyes and subsequent histological examination confirmed no detectable CNS10-NPC, demonstrating that bleb formation prior to cell delivery is required. To use a 1-cannula approach for both bleb creation and subsequent cell injection, we evaluated bleb creation using air, rather than transplantation media. A single cannula was first loaded with cells, followed by 25 µL of air. Using the automatic injection system, a 25 µL air bubble was injected in order to create a subretinal bleb, followed by cell injection. This was performed bilaterally at a non-survival timepoint (n = 1) and at a 7 day survival timepoint (n = 2). Surgical microscopic evaluation showed no cell reflux, presumably due to the air bubble preventing the collapse of the bleb. Histological examination revealed that, while one 7 day survival pig had no detectable cells, the non-survival pig and additional 7 day survival pig had CNS10-NPC in both injected eyes.

**Fig. 5:**
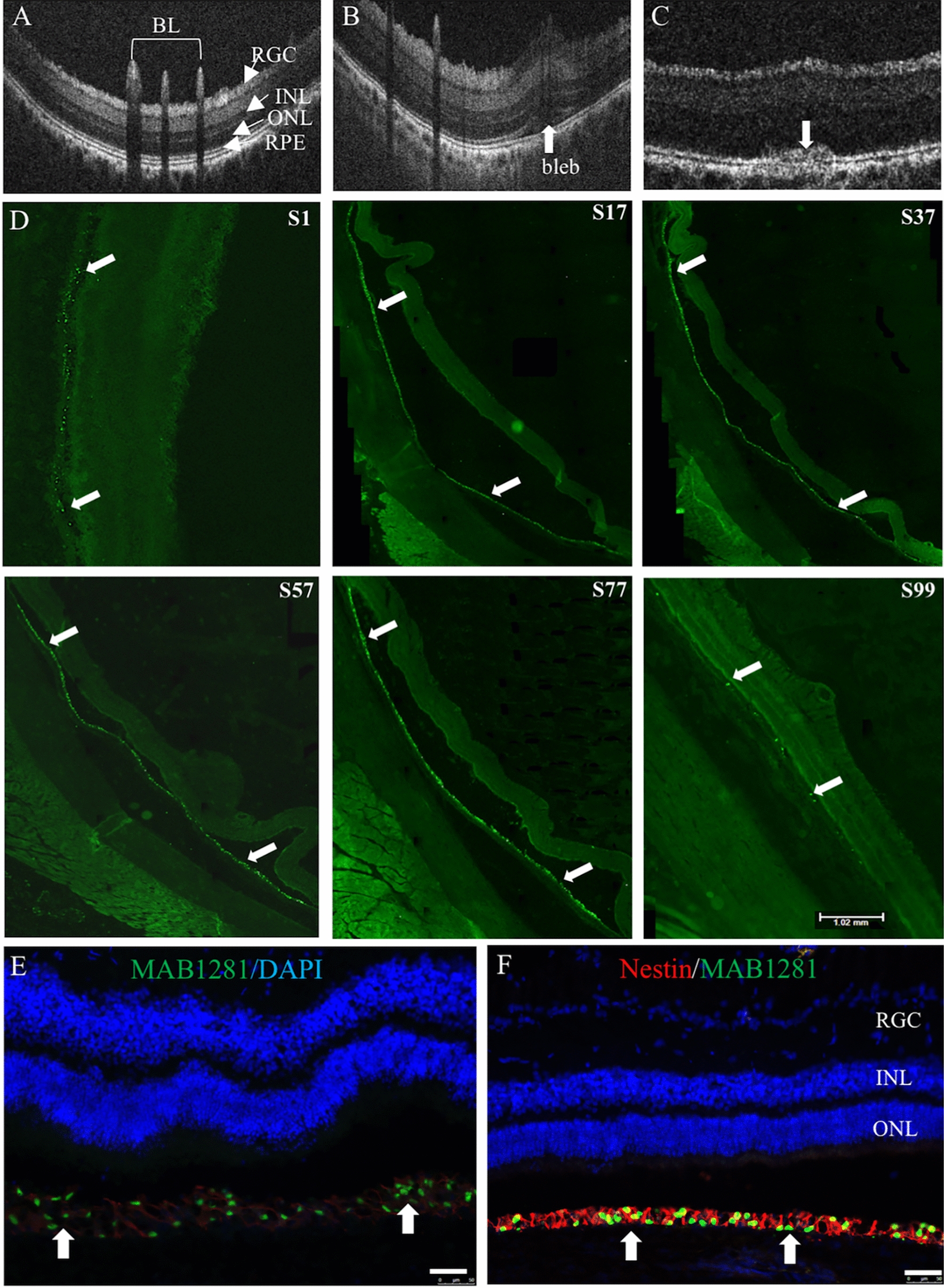
Donor cell survival and distribution in a large animal model. Yucatan minipig received a single subretinal injection of CNS10-NPC (10,000 cells/μl, 50 μl in total). (**A**–**C**) Fundus images were taken 30 min before, and then 30 min and 1 week post-injection by SD-OCT; **B** showed retinal detachment (Arrow pointing to the bleb) and **C** showed lumps in the subretinal space (arrow). (**D**) Retinal sections stained with human nuclear marker MAB1281 showed extensive donor cell distribution from section (S1) to S99, arrows pointing to MAB1281-positive donor cells one week after cell injection. (**E** and **F**) Confocal images show MAB1281-positive donor cells in the subretinal space (Arrows in **E**) and double positive staining for MAB1281 and the neural progenitor cell marker nestin (Arrows in **F**). Scale bars = 50 μm in **E** and **F**, 1.02 mm in **D S99**. *BL* blood vessels, *INL* inner nuclear layer, *IPL*, inner plexiform layer, *ONL* outer nuclear layer, *OPL* outer plexiform layer, *RGC* ganglion cell layer, *RPE* retinal pigment epithelium

SD-OCT and immunohistochemistry were performed to assess CNS10-NPC survival in the minipig. SD-OCT images taken 30 min before and then 30 min and 7 days after cell injection show that a bleb was clearly visible at 30 min after injection, and a small cell clump was seen at 7 days (Fig. 5A–C). Immunohistochemistry with a human-specific MAB1281 demonstrated CNS10-NPC formed a layer of cells and distributed from section (S) S1 to S99 at 7 days post-injection (Fig. 5D). Confocal images showed that CNS10-NPC survived and remained as nestin-positive neural progenitors (Fig. 5E and F). Collectively, this large animal model demonstrated that bleb formation prior to cell infusion with a single retinotomy and 1-cannula automatic injection system were optimal for the delivery of CNS10-NPC.

## Discussion

This study shows that both research- and clinical-grade NPC provide photoreceptor and vision preservation following a single subretinal injection into the RCS rat model of RP. Grafted CNS10-NPC remained as neural progenitors or matured into astrocytes and did not differentiate into retinal cells. CNS10-NPC offered photoreceptor and vision preservation and survived up to 6 months in the degenerative retinal environment, with no evidence of unwanted cell growth or pathology even at the maximum feasible dose. Finally, CNS10-NPC survived and distributed in the retina of Yucatan minipigs. This comprehensive study thus presents a path from laboratory discovery to an ongoing clinical trial.

Visual acuity measured by OKR showed no deterioration from P60 and P90 in current study, and significantly higher visual acuity at 6 months post-injection in the 60 K and high dose groups. Histological examination revealed that preservation of photoreceptors and cones correlated with the donor cell distribution, which supports the visual acuity data, since visual acuity only needs a small area with functional photoreceptors present to elicit a response. Our previous long-term study with NPC in the RCS rat provides further evidence by luminance threshold recording (LTR) from the superior colliculus [37]. The LTR clearly demonstrated that graft-protected area was reduced when the same animals were recorded over time. However, the central graft-protected area remains sensitive to light stimuli.

RCS rats have a mutation in *MertK*, and similar mutations in *MERTK*, the human orthologue, have been found in patients with RP [55]. While the rat provides for a model of retinal degeneration to test efficacy, cell delivery needs to be assessed in a large animal model. As the Yucatan minipig eye size is comparable to the human, it was used to optimize the surgical methodology. The identified delivery system is now being used in our Phase 1/2a clinical trial (NCT04284293) of transplanting CNS10-NPC into the subretinal space of RP patients.

Preclinical and clinical studies have shown that a single subretinal delivery of cells or gene therapy only affects one-third of the retinal area, leaving a majority of the retina to continue progressive retinal degeneration [17, 18, 27, 56]. These limitations of cell therapy could explain the reduced ERG response overtime, as this test measures the average of overall retinal response to light stimuli. Even though neural progenitors show migration in the rodent and minipig, it is possible that multiple cell injections to cover a majority of the retinal area may be needed to provide a broader therapy. A limitation of this study is only early timepoints for an early stage of retinal degeneration was tested. To be more clinically relevant, later timepoints of retinal degeneration should be tested to examine donor cell survival and efficacy in a more degenerative retinal environment. It is well-known that the degenerative retinal environment becomes hostile due to inflammation, oxidative stress and trophic factor deprivation. Since most patients presenting to the clinic already have substantial photoreceptor loss, stem/progenitor cell-based treatments may require additional trophic support or anti-inflammatory treatments to improve the degenerative retinal environment and support the function of cell therapeutics. Trophic factors have been shown to be effective in slowing down retinal degeneration, particularly, glial cell line-derived neurotrophic factor (GDNF) can provide direct protection of photoreceptors, which express specific GDNF receptors [57] and can indirectly protect photoreceptors via retinal Müller glia [58]. Furthermore, GDNF can increase phagocytosis by RPE cells in AMD [59]. We have already genetically engineered CNS10-NPC to stably secrete GDNF, and expanded and banked this cell line (termed, CNS10-NPC-GDNF) as a clinical product [46]. CNS10-NPC-GDNF has demonstrated safety in a recent Phase 1/2a clinical trial for amyotrophic lateral sclerosis (ALS) after delivery to the lumbar spinal cord (NCT02943850) [60], and is being currently delivered to the motor cortex of ALS patients (NCT05306457). This combined cell and trophic factor therapy may enhance vision protection in treating RP.

Fetal retinal progenitor cells used by ReNeuron appear to mainly stay as a clump at the injection site in a minipig model [36]. In contrast, our NPC product has been shown to migrate long distances from the injection site in multiple animal models [31, 37, 39, 61] including rodents, Yucatan minipigs, and nonhuman primates. Furthermore, neural progenitor/stem cells derived from the central nervous system and human pluripotent stem cells have shown neuroprotective effects in animal models for several disorders including ALS, Huntington’s disease, dementia, stroke, spinal cord injury and retinal degeneration [35, 62–68]

As with ReNeuron, the NPC are directly injected into the subretinal space, which is in stark contrast to JCyte trials that inject cells into the vitreous humor, with no published data showing that cells migrate to the photoceptor layer to protect/replace dying photoreceptors. While retinal progenitor cells aim to replace lost cells, the published preclinical characterization shows cell survival and efficacy in the RCS rat model, yet no evidence of grafted retinal progenitor cell differentiation into retinal cell types [69]. The ReNeuron Phase 2a trial has been halted due, in part, to complications from surgery. Our NPC have been shown to protect host photoreceptors and improve the retinal environment. This is through various mechanisms of action, including to regulate the immune response; promote antioxidant effects; release pro-survival factors [32, 40]; prevent outer segment accumulation by phagocytosis [38] and release protective trophic factors [31].

The RCS rat is a homolog model for RP, and it has also been widely used as an AMD-like model due to the primary RPE defect, although the model does not fully recapitulate AMD pathophysiology. We have performed several preclinical studies in this model that have led to clinical trials using stem cell therapy for AMD [16, 34, 35]. However, clinical reports from RPE replacement therapy for AMD have shown inconsistent efficacy [70, 71]. The main obstacle is that grafted RPE cells failed to integrate onto the Bruch’s membrane, likely because the degenerating membrane is not a supportive milieu for new RPE grafts. An alternative cell type that can protect photoreceptors and vision without needing to attach to the Bruch’s membrane may thus be a viable strategy. Our cell product does not need to attach to the membrane and can reduce the burden of ailing RPE cells by both phagocytosing outer segments and providing trophic support to the degenerative retinal environment [31, 32, 38]. As such, the CNS10-NPC clinical product provides a promising treatment to delay progressive retinal degeneration in both RP and AMD. This current study, along with extensive prior work, provides the bench-to-bedside path for a clinical product to reach a Phase 1/2a clinical trial for the treatment of both RP and AMD patients.

### Supplementary Information

**Additional file 1: Table S1.** Cohorts in rodent studies.

**Additional file 2: Table S2.** List of antibodies used in this study.

**Additional file 3: Table S3.** CNS10-NPC characterization. **S3A**: CNS10-NPC survival at P90. **S3B**: Detection of CNS10-NPC at different times. **S3C**: Incidence of CNS10-NPC division in treated RCS rats.

**Additional file 4: Fig S1.** hNPC and CNS10-NPC preserve vision in a dose-response fashion. **A** and **B** Optokinetic response (OKR) shows that hNPC-treated groups have significantly higher spatial visual acuity in the optimal dose (60 K) compared with other groups at both P60 and P90 timepoints. There is no significant difference between the optimal dose and higher dose groups (n=3 for vehicle treated, n=6 for 6 K dose, n=9 for 20 K-400 K doses, n=45 for untreated eyes). **C** and **D** Photopic electroretinography (ERG) shows that the optimal dose offered significantly higher b-wave amplitude compared with low dose and control groups, with no significant difference among the three high doses. **E** Spectral Domain Optical Coherence Tomography (SD-OCT) was performed at P60 and P90 following cell or vehicle injection at P21-23. At P60, ONL is visible from all the groups, but is clearly thicker in the 60K-400K hNPC groups. Lumps (Arrows showing cells in 200 K and 400 K were detected in retinas with 60 K or higher treatment at P60 associated with thicker ONL, which were largely flatten out at P90). The ONL in lower dose and controls was no longer visible, while in 60 K or higher dose treated groups, ONL was clearly visible at P90. Scale bar = 400 µm. Data are represented as mean ± SEM. One-way ANOVA with Tukey’s test was used for multiple comparisons. *p<0.05; ** p<0.01; *** p<0.0001; ns: no significance.

**Additional file 5: Fig S2.** Photoreceptor protection with hNPC treatment is dose-dependent. **A** Retinal sections stained with cresyl violet show consistent photoreceptor protection with 20 K dose, while retinas from 60 K–400 K groups have 6-8 layers of photoreceptors (Arrows) associated with donor cell distribution (Triangles). **B** The length of preserved ONL (more than 2 nuclei thickness) against the whole retinal length measured by ImageJ shows 20 K dose and above had significantly better ONL protection than other groups, with no significant difference among 60 K and 400 K groups. **C** Retinal section with 60 K treatment stained with cone arrestin antibody and human marker Stem 121 reveal cone profile with inner and outer segments, and cone pedicles (Triangles) were preserved compared with untreated retina. a–b’ are high power images of the outlines. Retinal whole-mount stained with cone arrestin and counterstained with DAPI show preserved cones with high density with 60K treatment compared with degenerating cones in untreated retina. c’ and d’ are high power images of the outlines showing cones. Scale bars = 50 μm in **B** and 25 μm in **C**. Data are represented as mean ± SEM. One-way ANOVA with Tukey’s test was used for multiple comparisons. *** p<0.001.* INL* inner nuclear layer, *ONL* outer nuclear layer, *IS *Inner segments, *OS* Outer segments.

**Additional file 6: Fig S3.** GLP study animal physiology. Body weight of male and female rats, as well as weight from brains, hearts, lungs, kidneys, lymph nodes and spleens from GLP study. There is no difference among cell-treated groups and vehicle control group.

## Data Availability

The datasets used and/or analysed during the current study are available from the corresponding authors upon reasonable request.

## Abbreviations

AMD: Age-related macular degeneration;
CNS10-NPC: Clinical-grade hNPC
ERG: Electroretinography
GLP: Good laboratory practice
GMP: Good manufacturing practice
hNPC: Human neural progenitor cell
IND: Investigational new drug
OKR: Optokinetic response
ONL: Outer nuclear layer
RP: Retinitis pigmentosa
RCS: Royal college of surgeons
RPE: Retinal pigment epithelium
SD-OCT: Spectral domain optical coherence tomography
