# Supplementary figures and images for: GMP-grade human neural progenitors delivered subretinally protect vision in rat model of retinal degeneration and survive in minipigs
Source: J Transl Med. 2023 Sep 25;21:650. doi: 10.1186/s12967-023-04501-z (PMC10519102; doi:10.1186/s12967-023-04501-z)

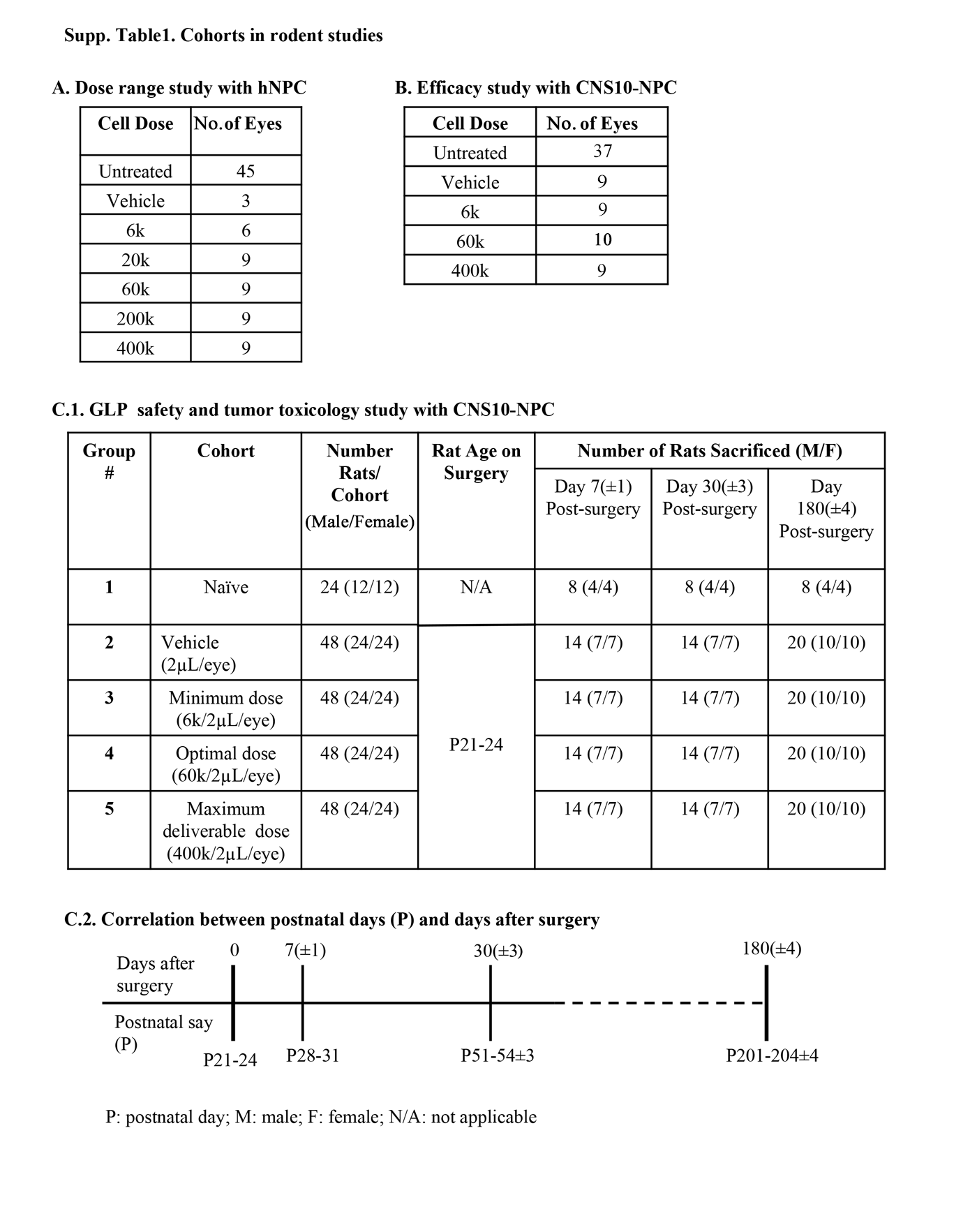

Supplement: Supplementary file 1 — Additional file 1: Table S1. Cohorts in rodent studies. [file 12967_2023_4501_MOESM1_ESM.tif]

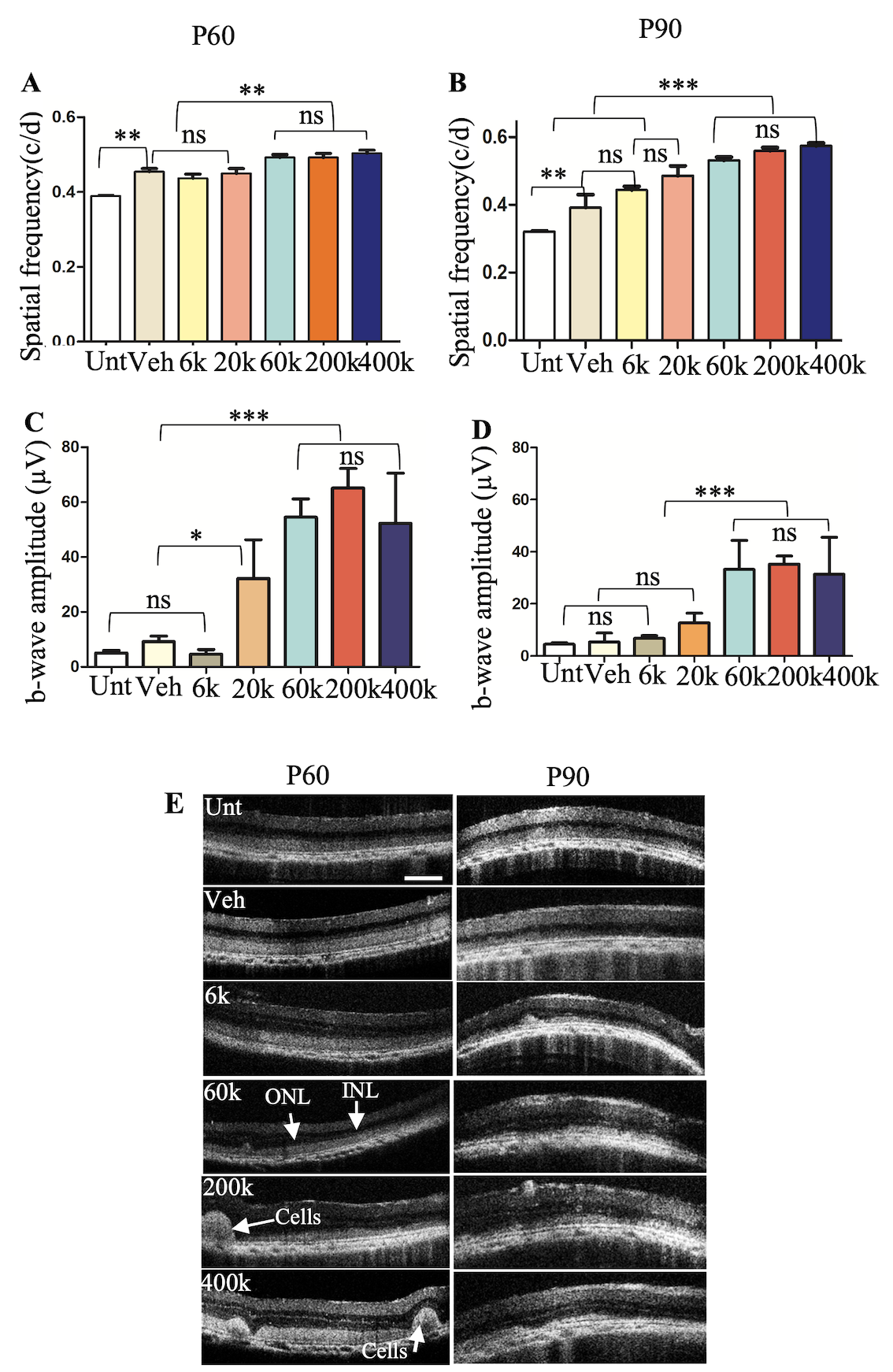

Supplement: Supplementary file 4 — Additional file 4: Fig S1. hNPC and CNS10-NPC preserve vision in a dose-response fashion. A and B Optokinetic response (OKR) shows that hNPC-treated groups have significantly higher spatial visual acuity in the optimal dose (60 K) compared with other groups at both P60 and P90 timepoints. There is no significant difference between the optimal dose and higher dose groups (n=3 for vehicle treated, n=6 for 6 K dose, n=9 for 20 K-400 K doses, n=45 for untreated eyes). C and D Photopic electroretinography (ERG) shows that the optimal dose offered significantly higher b-wave amplitude compared with low dose and control groups, with no significant difference among the three high doses. E Spectral Domain Optical Coherence Tomography (SD-OCT) was performed at P60 and P90 following cell or vehicle injection at P21-23. At P60, ONL is visible from all the groups, but is clearly thicker in the 60K-400K hNPC groups. Lumps (Arrows showing cells in 200 K and 400 K were detected in retinas with 60 K or higher treatment at P60 associated with thicker ONL, which were largely flatten out at P90). The ONL in lower dose and controls was no longer visible, while in 60 K or higher dose treated groups, ONL was clearly visible at P90. Scale bar = 400 µm. Data are represented as mean ± SEM. One-way ANOVA with Tukey’s test was used for multiple comparisons. *p<0.05; ** p<0.01; *** p<0.0001; ns: no significance. [file 12967_2023_4501_MOESM4_ESM.tif]

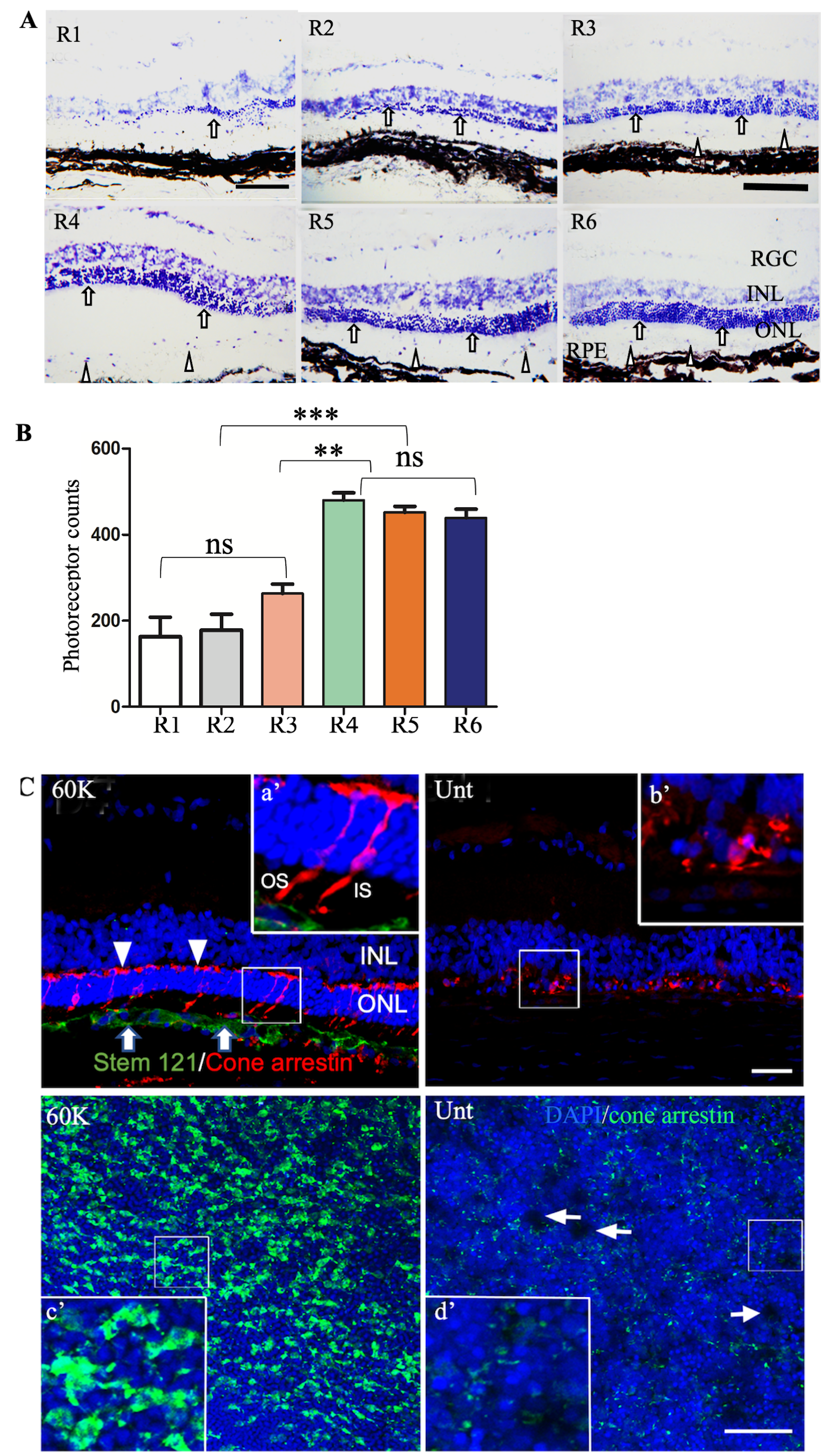

Supplement: Supplementary file 5 — Additional file 5: Fig S2. Photoreceptor protection with hNPC treatment is dose-dependent. A Retinal sections stained with cresyl violet show consistent photoreceptor protection with 20 K dose, while retinas from 60 K–400 K groups have 6-8 layers of photoreceptors (Arrows) associated with donor cell distribution (Triangles). B The length of preserved ONL (more than 2 nuclei thickness) against the whole retinal length measured by ImageJ shows 20 K dose and above had significantly better ONL protection than other groups, with no significant difference among 60 K and 400 K groups. C Retinal section with 60 K treatment stained with cone arrestin antibody and human marker Stem 121 reveal cone profile with inner and outer segments, and cone pedicles (Triangles) were preserved compared with untreated retina. a–b’ are high power images of the outlines. Retinal whole-mount stained with cone arrestin and counterstained with DAPI show preserved cones with high density with 60K treatment compared with degenerating cones in untreated retina. c’ and d’ are high power images of the outlines showing cones. Scale bars = 50 μm in B and 25 μm in C. Data are represented as mean ± SEM. One-way ANOVA with Tukey’s test was used for multiple comparisons. *** p<0.001. INL inner nuclear layer, ONL outer nuclear layer, IS Inner segments, OS Outer segments. [file 12967_2023_4501_MOESM5_ESM.tif]
