# Supplementary material for: GMP-grade human neural progenitors delivered subretinally protect vision in rat model of retinal degeneration and survive in minipigs
Source: J Transl Med. 2023 Sep 25;21:650. doi: 10.1186/s12967-023-04501-z (PMC10519102; doi:10.1186/s12967-023-04501-z)
Supplement: Supplementary file 2 — Additional file 2: Table S2. List of antibodies used in this study. [file 12967_2023_4501_MOESM2_ESM.pdf]

Additional file Table 2. Antibodies used in the study

Name	Dilution	Manufacturer
Cone arrestin	1:5000	Millipore, Billerica, MA
GFAP	1:1000	Signa, St. Louis, MO
Ki67	1:100	ThermoScientific
MAB1281	1:300	Millipore, Billerica, MA
Nestin	1:10000	Millipore, Billerica, MA
PKCα	1:8000	Sigma, St. Louis, MO
Recoverin	1:2000	Millipore, Billerica, MA
Stem 121	1:300	Stem Cells, Newark, CA
Stem 101	1:100	Stem cell Inc., Cambridge
Stem 123	1:3000	Stem Cells, Newark, CA
Synaptophysin	1:1000	Millipore, Billerica, MA
