# Supplementary material for: GMP-grade human neural progenitors delivered subretinally protect vision in rat model of retinal degeneration and survive in minipigs
Source: J Transl Med. 2023 Sep 25;21:650. doi: 10.1186/s12967-023-04501-z (PMC10519102; doi:10.1186/s12967-023-04501-z)
Supplement: Supplementary file 3 — Additional file 3: Table S3. CNS10-NPC characterization. S3A: CNS10-NPC survival at P90. S3B: Detection of CNS10-NPC at different times. S3C: Incidence of CNS10-NPC division in treated RCS rats. [file 12967_2023_4501_MOESM3_ESM.pdf]

Additional file Table 3A: Donor cell survival at P90

Group	Cells in subretinal space	Cells in inner retina	Total cell number	Total cell injection	Cell survival(%)
G1	0	0	0	0	0
G2	0	0	0	6,000	0
G3	4400 ± 1339	144 ± 80	4545 ± 1383	60,000	7.6 ± 2.3
G4	25274 ± 3936	1305 ± 187	26579 ± 4093	400,000	6.6 ± 1.0

Additional file Table 3B: Detection of CNS10-NPC at different time

Group	Day	Total rats assessed	Common Staining	Uncommon Staining	No Staining
3 – 6,000 CNS10-NPC	7	14	1	10	3
	30	14	2	4	8
	180	16	0	1	15
4 – 60,000 CNS10-NPC	7	14	9	5	0
	30	14	9	2	3
	180	20	2	8	10
5 – 400,000 CNS10-NPC	7	14	13	1	0
	30	14	13	1	0
	180	15	11	2	2

Additional file Table 3C: Incidence of CNS10-NPC division in treated retina

Dose	Days post-surgery	Total rats assessed	Common staining	Uncommon staining	No staining
60K CNS10-NPC	7	6	0	4	2
	30	6	0	3	3
	180	7	0	0	7
