# Supplementary material for: GMP-grade human neural progenitors delivered subretinally protect vision in rat model of retinal degeneration and survive in minipigs
Source: J Transl Med. 2023 Sep 25;21:650. doi: 10.1186/s12967-023-04501-z (PMC10519102; doi:10.1186/s12967-023-04501-z)
Supplement: Supplementary file 6 — Additional file 6: Fig S3. GLP study animal physiology. Body weight of male and female rats, as well as weight from brains, hearts, lungs, kidneys, lymph nodes and spleens from GLP study. There is no difference among cell-treated groups and vehicle control group. [file 12967_2023_4501_MOESM6_ESM.pdf]

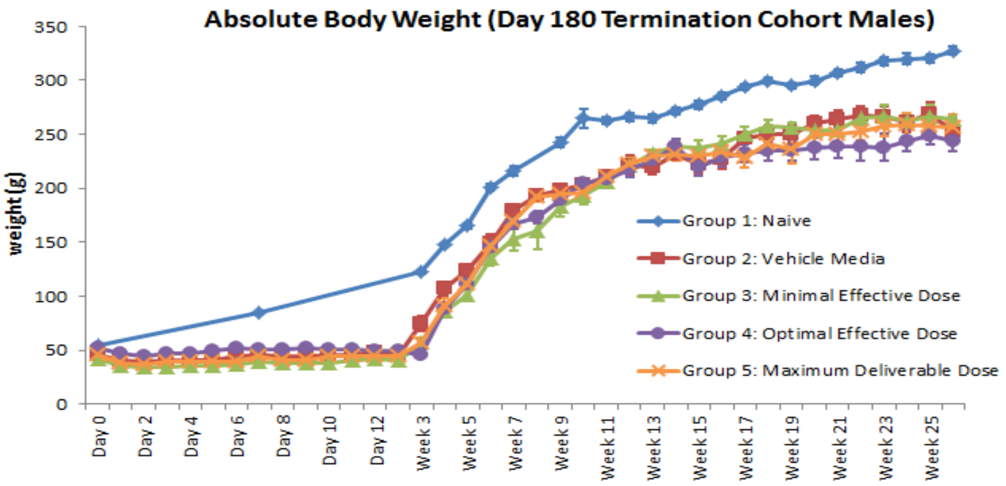
Absolute Body Weight (Day 180 Termination Cohort Males)
weight (g)
350
300
250
200
150
100
50
0
Day 0
Day 2
Day 4
Day 6
Day 8
Day 10
Day 12
Week 3
Week 5
Week 7
Week 9
Week 11
Week 13
Week 15
Week 17
Week 19
Week 21
Week 23
Week 25
Group 1: Naive
Group 2: Vehicle Media
Group 3: Minimal Effective Dose
Group 4: Optimal Effective Dose
Group 5: Maximum Deliverable Dose

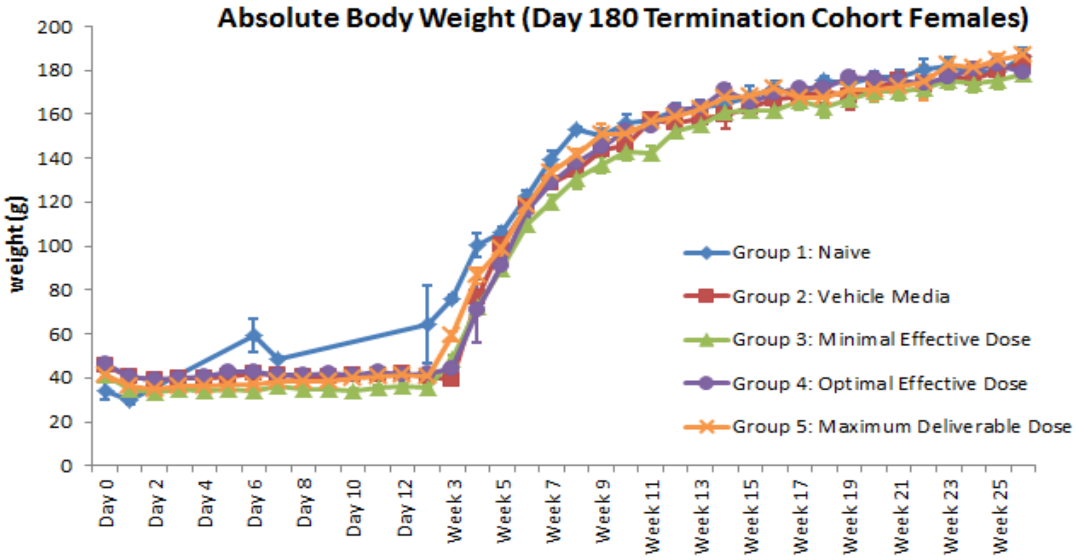
Absolute Body Weight (Day 180 Termination Cohort Females)
weight (g)
200
180
160
140
120
100
80
60
40
20
0
Day 0
Day 2
Day 4
Day 6
Day 8
Day 10
Day 12
Week 3
Week 5
Week 7
Week 9
Week 11
Week 13
Week 15
Week 17
Week 19
Week 21
Week 23
Week 25
Group 1: Naive
Group 2: Vehicle Media
Group 3: Minimal Effective Dose
Group 4: Optimal Effective Dose
Group 5: Maximum Deliverable Dose

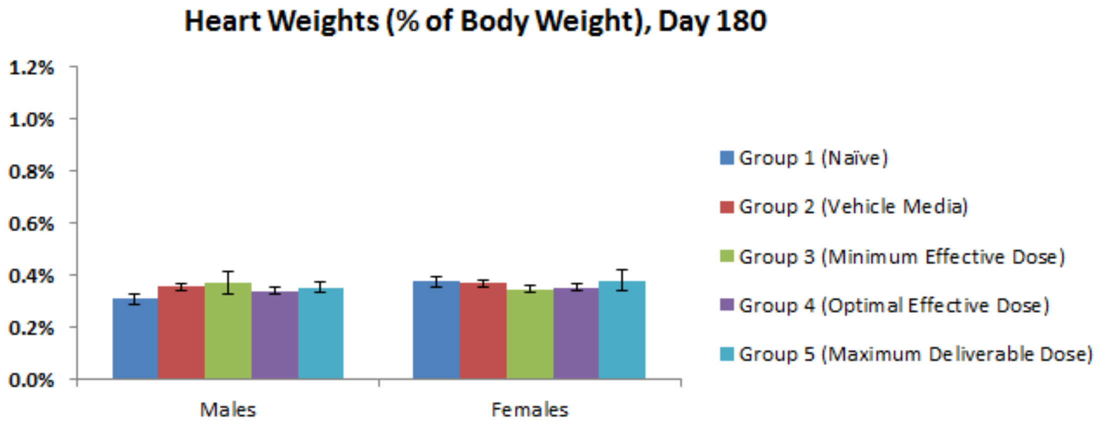
Heart Weights (% of Body Weight), Day 180
1.2%
1.0%
0.8%
0.6%
0.4%
0.2%
0.0%
Males
Females
Group 1 (Naïve)
Group 2 (Vehicle Media)
Group 3 (Minimum Effective Dose)
Group 4 (Optimal Effective Dose)
Group 5 (Maximum Deliverable Dose)

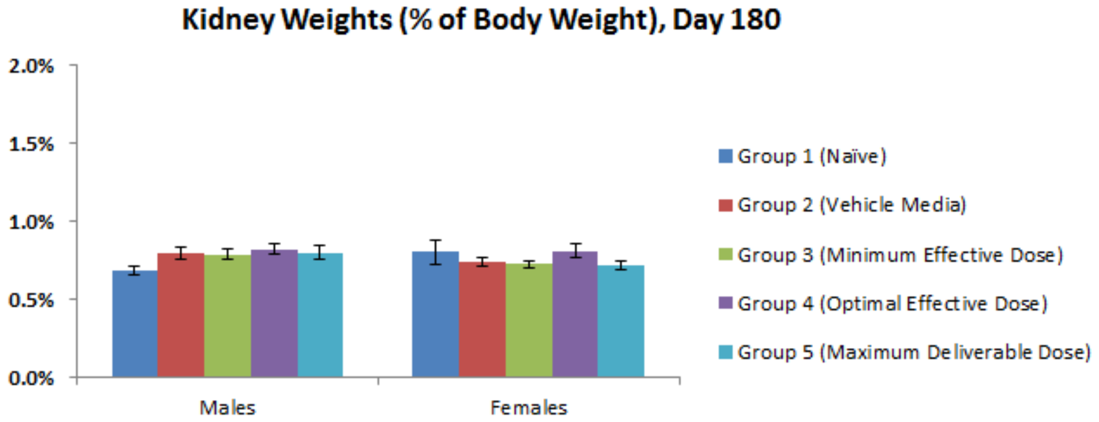
Kidney Weights (% of Body Weight), Day 180
2.0%
1.5%
1.0%
0.5%
0.0%
Males
Females
Group 1 (Naïve)
Group 2 (Vehicle Media)
Group 3 (Minimum Effective Dose)
Group 4 (Optimal Effective Dose)
Group 5 (Maximum Deliverable Dose)

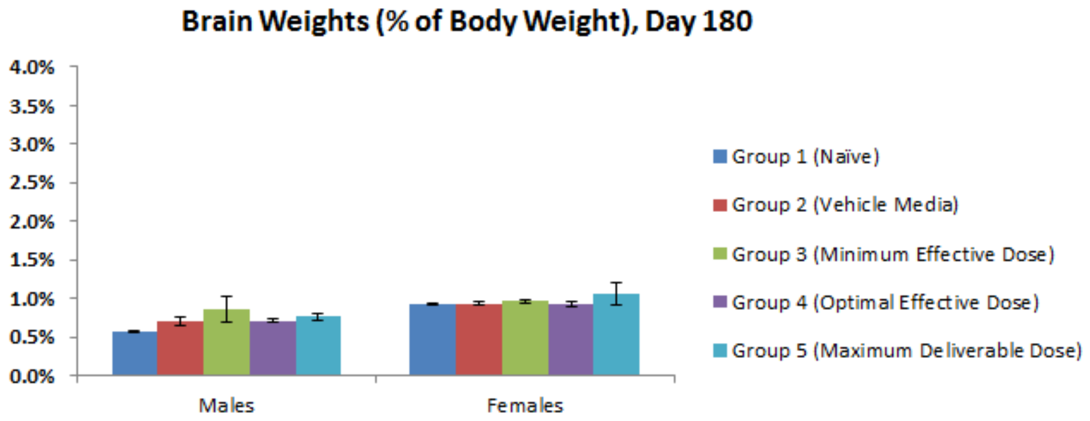
Brain Weights (% of Body Weight), Day 180
4.0%
3.5%
3.0%
2.5%
2.0%
1.5%
1.0%
0.5%
0.0%
Males
Females
Group 1 (Naïve)
Group 2 (Vehicle Media)
Group 3 (Minimum Effective Dose)
Group 4 (Optimal Effective Dose)
Group 5 (Maximum Deliverable Dose)

Liver Weights (% of Body Weight), Day 180

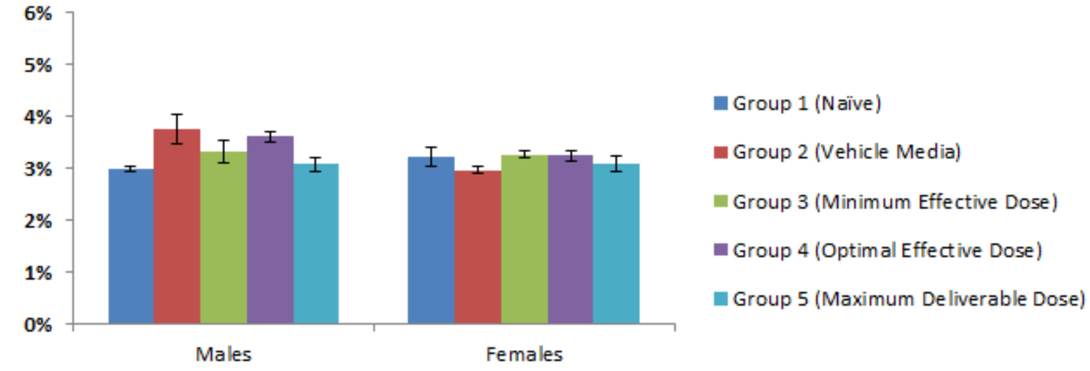

Lung Weights (% of Body Weight), Day 180

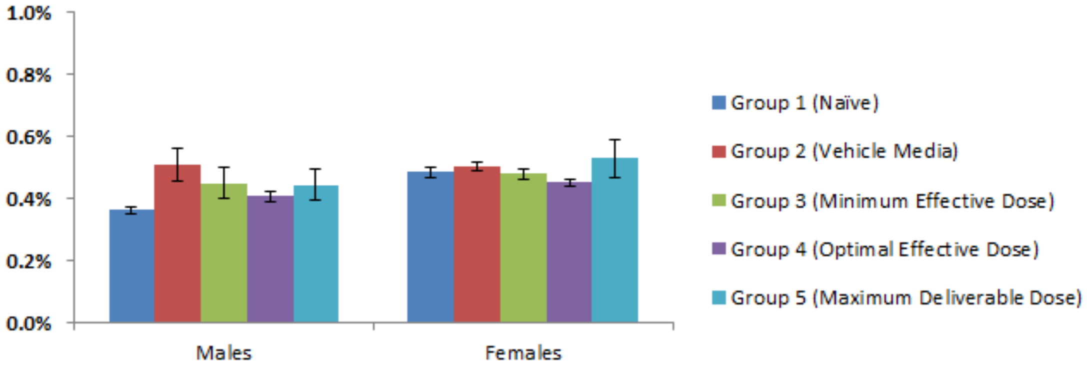

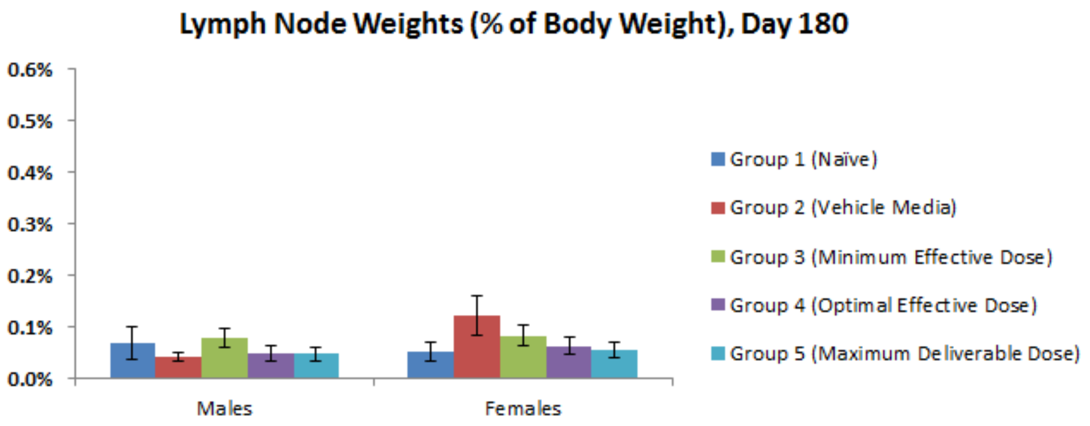
Lymph Node Weights (% of Body Weight), Day 180
0.6%
0.5%
0.4%
0.3%
0.2%
0.1%
0.0%
Males
Females
Group 1 (Naïve)
Group 2 (Vehicle Media)
Group 3 (Minimum Effective Dose)
Group 4 (Optimal Effective Dose)
Group 5 (Maximum Deliverable Dose)

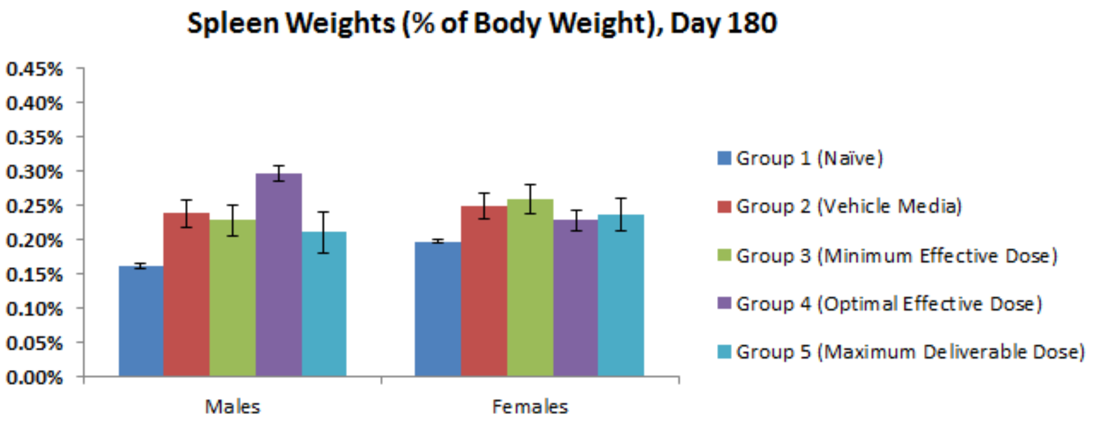
Spleen Weights (% of Body Weight), Day 180
0.45%
0.40%
0.35%
0.30%
0.25%
0.20%
0.15%
0.10%
0.05%
0.00%
Males
Females
Group 1 (Naïve)
Group 2 (Vehicle Media)
Group 3 (Minimum Effective Dose)
Group 4 (Optimal Effective Dose)
Group 5 (Maximum Deliverable Dose)
